# Investigating the Evolution of Student Attitudes toward Science in a General Chemistry Course Using Latent Class and Latent Transition Analysis

**DOI:** 10.1021/acs.jchemed.4c01247

**Published:** 2025-04-22

**Authors:** Oluwatobi O. Odeleye, Oluwaseun D. Agunbiade, Adam Garber, Karen Nylund-Gibson

**Affiliations:** † Department of Chemistry, West Virginia University, Morgantown, West Virginia 26505, United States; ‡ University of California − Santa Barbara, Santa Barbara, California 93106, United States

**Keywords:** General Chemistry, Attitudes, Mixture Modeling, Latent Transition Analysis, Chemistry Education Research

## Abstract

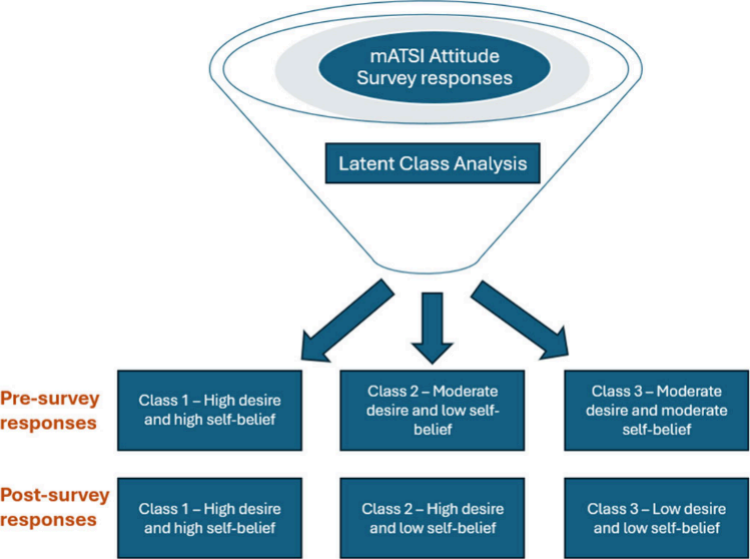

As science, technology, engineering, and mathematics (STEM) education researchers continue to explore ways to increase college student persistence in STEM fields, the affective domain (e.g., attitudes, perceptions, and self-efficacy) stands out as an area that can significantly impact these efforts. Latent class analysis (LCA) and latent transition analysis (LTA) are mixture modeling approaches that take a person-centered approach to quantitative research, which can help us to further our efforts to diversify STEM fields. This study seeks to use LCA and LTA to investigate how students’ attitudes toward science in general chemistry evolve over a semester. Using the *Modified Attitudes toward Science Inventory* (mATSI), we grouped students based on their responses to pre- and postsurvey items from the mATSI. We found three distinct groups (classes) of students at the beginning of the semester: (i) students with strong desires to pursue science fields and high self-belief in their abilities to do well in science courses (high–high), (ii) students with moderate desires and low self-belief (mod-low), and (iii) students with moderate desires to pursue science fields and moderate self-belief (mod-mod). Over the course of the semester, these 3 groups evolved into (a) high desires and high self-belief (high–high), (b) high desires and low self-belief (high-low), and (c) low desires and low self-belief (low-low). At the beginning of the semester, about 80% of the participants were classified in the high–high group with the remaining 20% categorized into the other two groups; however, by the end of the semester, about 70% were in the high–high group, with 30% distributed across the other two groups. Using LTA and exploring the characteristics of the student groups, we found that in groups where female and second-year students were overrepresented, male and first-year students tended to be underrepresented and vice versa. For example, female and second-year students were overrepresented in groups more likely to leave the general chemistry course with lower desires and self-belief, while male and first-year students were overrepresented in groups more likely to leave general chemistry with higher desires and self-belief Using the LCA approach, we were able to explore groups (e.g., “high-low” and “low-low”) that tend to get swallowed up by the noise of the majority (in this case, the “high–high” group). We hope the findings from this study encourage equity-based researchers to continue to think about how they approach quantitative data to give a voice to participant groups that may sometimes be hidden under the guise of not having enough statistical significance/power.

## Introduction

In the bustling realm of science education, researchers and educators have long sought to explore the complex factors influencing student learning performance, attitudes/perceptions, persistence, and achievement in chemistry education.^1,2^ Students’ attitudes toward general chemistry courses are an integral component of undergraduate education significantly influencing the students’ learning processes, performance, and persistence in STEM fields.^3,4^ According to Ing and Nylund-Gibson,^5^ exploring students’ attitudes beyond achievement and grades is essential because these attitudes can be influenced by various range of factors such as “who is teaching, what is being taught, and how it is being taught”.^5^ Therefore, focusing on what could influence their attitudes, interests, and self-efficacy toward chemistry and science education should be a substantial concern. Perceptions and attitudes are both terms that have been used interchangeably in discipline-based education research and are used interchangeably in this paper. However, most researchers would agree that these concepts are multidimensional.^6^ In their paper, van Aalderen-Smeets et al. define attitude generally as “a psychological tendency to evaluate an object in terms of favorable or unfavorable attribute dimensions such as good/bad or positive/negative”.^6^ They identified four main subdomains of personal attitudes toward science: cognition, affect, behavioral intention, and self-efficacy. They characterized the cognitive aspect of attitude as involving “the perceived relevance of science” to society and daily life, “perceived difficulty of science”, and “beliefs about gender differences in science”. Affect was described as “emotions or feelings related to science” and behavioral intention as behaviors or activities related to science in the individual’s daily life. Finally, they characterized self-efficacy as the individual’s “perceived level of capability or confidence” in completing a task that may be challenging. Based on van Aalderen-Smeets et al.’s characterizations of the various aspects of attitude,^6^ the Modified Attitudes Toward Science Inventory (mATSI) was the instrument used in this study, because had items that measured all four subdomains of attitude.

### Theoretical Framework

There are two theoretical frameworks that guide this study: self-efficacy and self-determination theory (SDT). Since self-efficacy is one of the subdomains this study seeks to investigate, Bandura’s self-efficacy theory, which suggests that an individual’s belief in their abilities can drive the decisions they make as well as their behaviors, is a framework that guides this study.^7,8^ This theory suggests that individuals with high self-efficacy will likely exhibit positive behaviors and desires toward science. The second framework that guides this study is the self-determination theory (SDT).^9,10^ The SDT postulates that social and cultural factors can influence an individual’s motivation and desires. Ryan and Deci highlight the three phases of motivation as (a) amotivation (no motivation), (b) extrinsic motivation (influenced by external forces) and, (c) intrinsic motivation (influence by personal/internal forces).^11^ This theory suggests that an individual’s desire (or lack thereof) to pursue science can be influenced by various factors.

## Latent Class Analysis (LCA) and Latent Transition Analysis (LTA)

One of the primary goals of chemistry education research is to foster students’ positive attitudes toward chemistry. Considerable research has explored student attitudes and perceptions with respect to their demographic characteristics: gender, socioeconomic status, race, and ethnicity.^12−15^ Traditional statistics methods like ANOVAs, and regression analyses, have been instrumental in building foundational knowledge in this area. In college chemistry, positive relationships have been found for first-year chemistry using correlation analysis^16^ and logistic regression analysis^17^ helping educators shape their teaching strategies.^18^ Xu et al. used multiple regression analyses to represent student achievement with their attitudes indicating a significant role in predicting student final achievement in a general chemistry course.^19^ These methods remain valuable and can be further enhanced by introducing person-centered approaches like Latent Class Analysis (LCA) and Latent Transition Analysis (LTA) to explore the evolving attitudes of students more deeply.

Latent Class Analysis (LCA) and Latent Transition Analysis (LTA) are specific mixture modeling techniques that can be used for quantitative data analysis.^20−22^ Mixture modeling, a person-centered statistical technique, is used to identify and understand complex patterns within a group.^23,24^ In educational research, mixture modeling reveals the underlying properties from the observable data. For instance, a researcher can use a traditional statistical approach to investigate the correlation between students’ extracurricular participation and academic performance. However, mixture modeling could allow researchers to understand the underlying reasons behind why students choose specific activities and the factors that influence these choices including patterns of behavior, and latent variables such as personal interests, social influences, skill development opportunities, and career aspirations^24−26^ Mixture modeling assumes that differences exist within individuals in any given population and seeks to draw out those differences.^27,28^ Latent class analysis classifies individuals with similar response patterns, identifying latent classes (or groups) and attempting to explain underlying hidden structures of observed data or variables under investigation.^29−35^ Using LCA enables the investigation of unobservable variables (i.e., latent variables) that contribute to subgroup formation, thereby integrating both observed (directly measured in the data they could be indicators, covariates, or distal) and latent variables.^36−38^

Latent Transition Analysis (LTA) is an extension of LCA that examines how these latent classes (or groups) change over time by combining cross-sectional measurement of categorical latent variables and longitudinal description of change.^39^ While LCA can uncover different groups of students with similar characteristics or needs at a given time point, LTA can shed light on how students’ attitudes or characteristics evolve by looking at changes over periods of time in the class (or groups)^40^ and potentially predicting the behavior transition with time.^41^

Several articles have been published regarding applications of LCA and LTA highlighting why these methods lend itself to equity-focused research in STEM education.^42−46^ However, there are drawbacks to using these methods. Some limitations of LCA and LTA include: (i) these methods are relatively new statistical analyses that may make data analysis and interpretation of results challenging, (ii) there is a cost barrier (financial and mental) to using these methods, and (iii) mixture modeling requires large data sets (exact numbers vary) to be confident in the results obtained.^20,29^

### Research Questions

This study sought to use person-centered approaches to explore the following research questions:

1. What groups of students, based on their perceptions of science, were found before and after taking a general chemistry class?
2. How do students transition between the identified groups (RQ 1), and what are the characteristics of the students that make up the groups?
3. What is the relationship between students’ perceptions and their final course grades?

## Method

This research was approved by the West Viginia Institutional Review Board (IRB) at the public, four-year, R1 university where the study took place (WVU Protocol #: 2207611431). The participants in this study were students enrolled in a first-semester general chemistry course (CHEM 1) in the Fall 2022 and Spring 2023 semesters. At this institution, CHEM 1 is taken by science and engineering majors and covers concepts like stoichiometry, properties of gages, thermochemistry, molecular structure and bonding, the periodic table, and atomic structure. At the beginning of the semester, an online survey, detailed in the Supporting Information, was administered to assess students’ attitudes toward science using the Modified Attitudes Toward Science Inventory (mATSI).^4,39,40^ The same survey was distributed at the end of the semester to assess any changes in attitudes. The postsurvey included additional questions to assess how students’ perceptions of science evolved over the semester and what factors they believe influenced this change. Both the pre- and postsurvey included two “attention check questions” to ensure students were paying attention as they were taking the survey. Responses of participants who did not select the correct prompts were not included in the data analysis. Students were recruited by emailing CHEM 1 course instructors asking them to share this research opportunity with their students. Instructors were encouraged to provide extra credit, up to 5% of the total course grade, for survey participation. Based on the IRB review, an optional assignment was created for students who wanted the extra credit opportunity but did not want to participate in the study. Most instructors provided some form of extra credit to incentivize participation (the highest amount of extra credit awarded was 2% of the total grade (1% for the presurvey and 1% for the postsurvey)); however, the extra credit provided did not significantly impact the students’ final grade (e.g., out of 100 total course points, students who completed the survey got 1 point for each survey they completed for a total of 2 points toward their overall grade). No interventions were applied to any class sections in this study.

### Participation Information

The target population for this study was students enrolled in CHEM 1 in the Fall 2022 (FA 22) and Spring 2023 (SP 23) semesters. The study participants were students enrolled in CHEM 1 (FA 22 and SP 23) who completed both the pre- and postsurveys sent out each semester. The presurvey was sent out to all students at the beginning of each semester, and the postsurvey was sent out 2 weeks before the end of each semester. Both surveys were conducted on Qualtrics and were open for 1 week. A total of 403 students participated in this study, and the demographic breakdown is shown in Table 1. Of the 403 participants, about 34% were engineering majors, 60% declared some science/health-related majors, and 6% had majors outside engineering and science/health (e.g., finance and political science). Due to the low sample sizes (a general rule of thumb is for the sample size to be at least 10% of the total population (n = 403 in this study)),^48^ we did not analyze data based on race/ethnicity. We also chose to look at data only for first- and second-year students due to the small sample size (less than 40 participants in each class) of the other student classes (i.e., “junior,” “senior,” and “other”).

**Table 1:** Participants’ Demographic Breakdown

	n	%
**Gender**		
Male	159	39%
Female	244	61%
**Year in School**		
First-year	280	69%
Sophomore	91	22%
Junior	25	6%
Senior	5	1%
Other	3	1%
**Semester**		
Fall 2022	189	47%
Spring 2023	214	53%
**First-Generation Status**		
First Generation	74	18%
Not First-Generation	329	82%

### Data Analysis Plan

Latent Class Analysis (LCA) was used to determine the different classes (groups of students) based on their responses to the mATSI survey. Of the 25 items on the mATSI instrument, responses to 9 of the 25 items were used to differentiate between the latent classes.^47,49^ The nine (9) items used created the most differentiation between the groups. For example, the first statement in the mATSI, “Science is useful in helping to solve the problems of everyday life”, was not a good item for differentiation because students across all groups were likely to agree with that statement and, as such, it was not included in the analysis. It should be noted that all negatively worded statements were reverse coded to ensure uniformity in understanding the results. More information on differentiation in latent class analysis (LCA) can be found in Nylund-Gibson and Choi’s “Ten frequently asked questions about latent class analysis”.^33^

A 5-point Likert scale was used (strongly agree to strongly disagree); however, for data analysis, the responses were categorized into two groups: strongly agree and agree responses were considered “yes” and strongly disagree and disagree responses were considered “no”. The “neither agree nor disagree” option was not included in the data analysis. Based on the system’s analysis, different groups of students emerged based on their responses to these questions. Latent transition analysis (LTA) was also used to determine how students transitioned from one class to another from the beginning to the end of the semester. We used MplusAutomation and R (Halliquist and Wiley)^50,51^ to estimate the different groups based on the responses and address research question 1. Research questions 2 and 3 were addressed by using descriptive analysis. Based on preliminary analysis of the demographic distribution and number of responses, we decided to explore class membership profiles based on gender (male, n = 159 or female, n = 244), year in school (first-year, n = 280, second-year, n = 91), and first-generation (FG) status (FG, n = 74 or non-FG, n = 329).

## Results and Discussion

### Research Question 1: What Groups Exist in Students’ Perceptions of Science before and after They Leave a General Chemistry Class?

**Figure 1:**
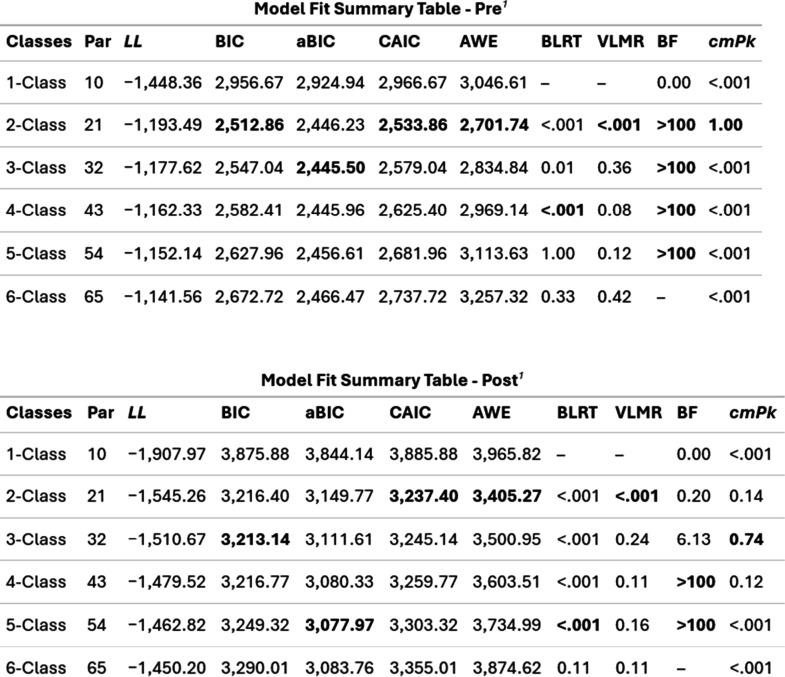
Fit statistics for pre- and postsurvey data. ^†^Note: PAR = parameters; *LL* = model log likelihood; BIC = Bayesian information criterion; aBIC = sample size adjusted BIC; CAIC = consistent Akaika information criterion; AWE = approximate weight of evidence criterion; BLRT = bootstrapped likelihood ratio test p-value; VLMR = Vuong-Lo-Mendell-Rubin adjusted likelihood ratio test p-value: *cmPk* = approximate correct model probability.

Research question 1 (RQ-1) was addressed by determining the number of latent classes by estimating a series of six models and analyzing model fit for both the pre- and postsurvey responses. To determine the number of groups for the LCA model solutions at both time points, each latent class’s theoretical meaning was carefully evaluated across the model solutions. It should be noted that because mixture modeling is a relatively newer field, the criteria to select the “best” solution (or number of groups/classes) is still evolving and experts in the field suggest using a theoretical lens in addition to the statistical criteria^34,52^ to select the “best solution”. Figure 1 shows the fit statistics criteria for the pre- and postsurvey data based on six different models. The information criteria indices (BIC, aBIC and CAIC in Figure 1) are fit indices where lower values indicate a better fit. For the likelihood-based tests (BLRT and VLMR in Figure 1), nonsignificant p-values indicate that adding a class to the model will not yield statistical improvement in the fit. For the Bayes Factor (BF in Figure 1), a BF less than 10, but greater than 3 indicates a moderate fit, while a BF greater than 10 indicates a strong fit. Finally, for the approximate correct model probability (cmPk in Figure 1), the largest number is the one that is selected. Using our knowledge of these fit indices, the statistical expertise of coauthors Garber and Nylund-Gibson, and the theoretical content knowledge of coauthor Odeleye, we compared the 2-class and 3-class solutions and concluded that the 3-class model provided additional interpretative value and coherence across pre- and postmodels relative to the 2-class solution. The codes that were used to run the statistical analyses in R are provided in the Supporting Information.

**Figure 2:**
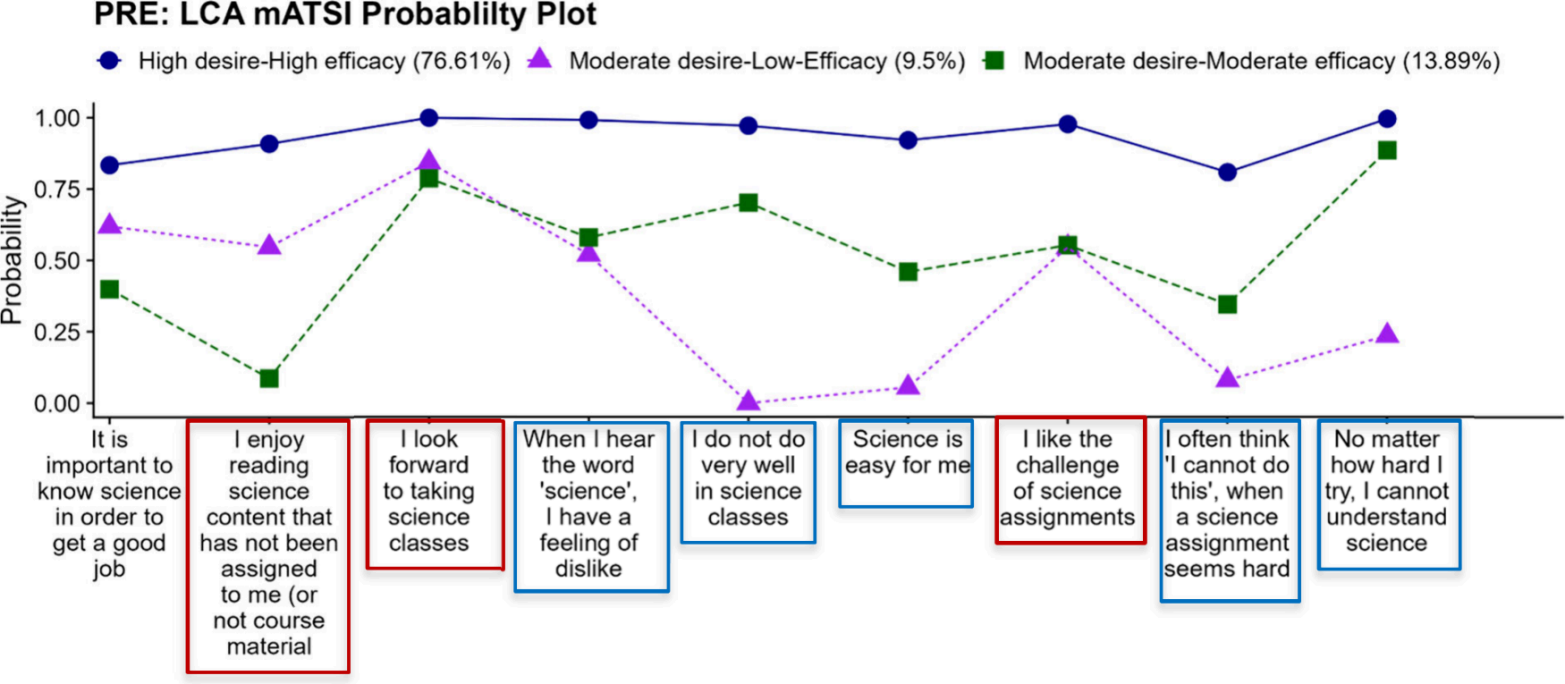
Probability plot of the groups that emerged based on latent class analysis (LCA) on the presurvey responses. (The items in the blue boxes indicate self-efficacy, while the items in the red boxes indicate desire.)

In the presurvey (Figure 2), the three identified groups found were as follows: Group 1 (77% of the total population) comprised *s*tudents with high desires to pursue careers in STEM fields and strong self-belief in their abilities (**high–high, HH**); Group 2 (10%) consisted of students with moderate desires to pursue STEM careers but low self-belief (**mod-low, ML**); Group 3 (14%) included students with moderate desires and moderate self-belief in their abilities (**mod-mod, MM**). Students in the high-desire, high-efficacy (HH) class were characterized by agreeing (above 75% likelihood) with all of the 9 mATSI indicators.^4,47^ For example, there was a 100% likelihood that these students looked forward to taking a science class, 93% likelihood that they believed science is easy for them, and 97% likelihood that they believed they would do well in science classes. Students in the moderate-desire, low-efficacy class (ML) were characterized by some level of agreement (around 75% likelihood) with items that had to do with their desires to take science classes and disagreement (less than 25% likelihood) with items that indicated a belief in their abilities to do well in science classes. For example, there was an 84% likelihood that students in this class looked forward to taking a science class, 7% likelihood they believe science is easy for them, and a 0% likelihood that these students believed they would do well in science classes. Students in the moderate-desire, moderate-efficacy (MM) class were characterized by some level of agreement (between 50 and 75%) with items that had to do with their desires to take science class and their belief in their abilities to do well in science classes. For example, there was a 79% likelihood that students in this class looked forward to taking a science class, 41% likelihood they believe science is easy for them, and a 70% likelihood that these students believed they would do well in science classes. These likelihood values (above 75%, around 75% and less than 25%) are arbitrary values based on the authors’ view and interpretation of the model).

**Figure 3:**
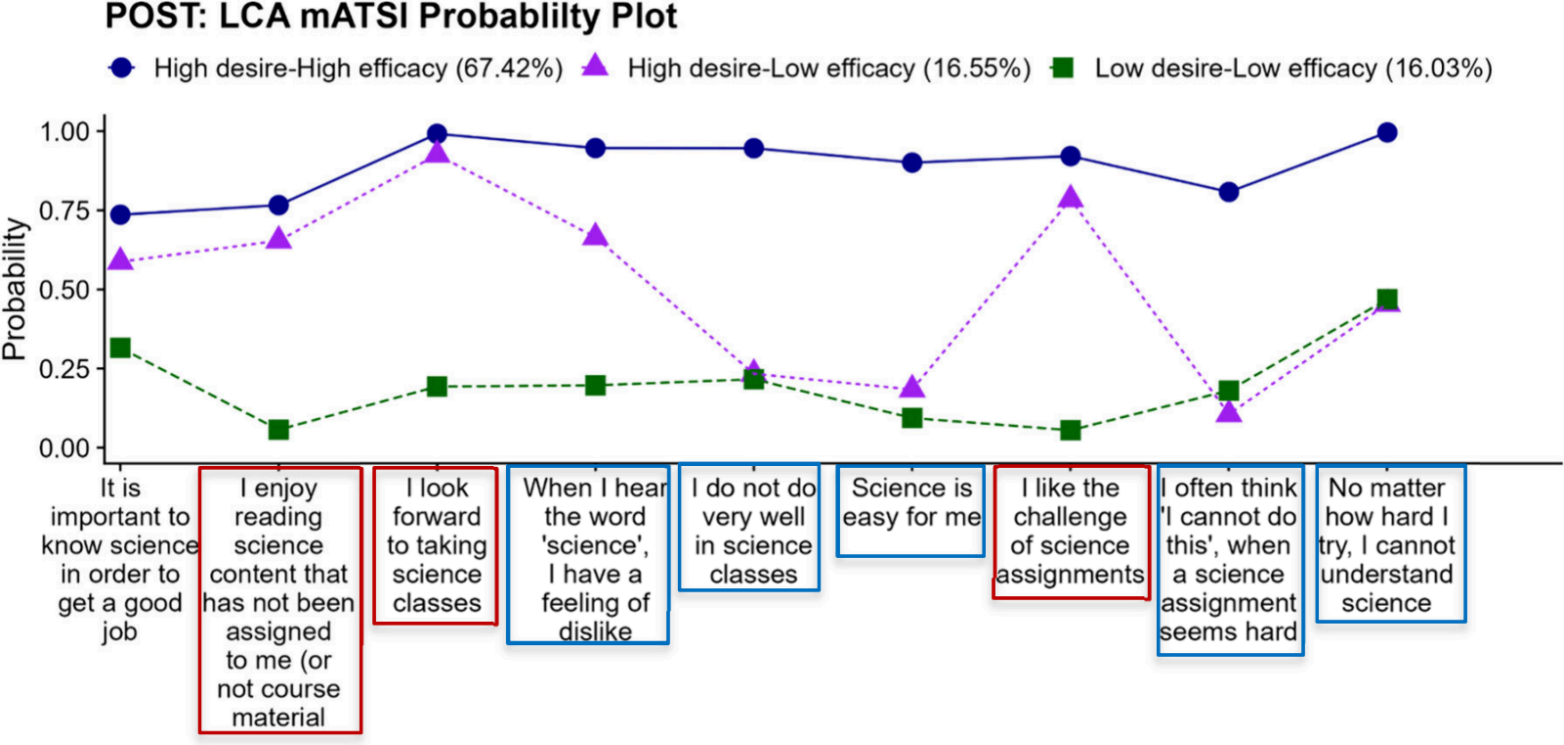
Probability plot of the groups that emerged based on latent class analysis (LCA) on the postsurvey responses. (The items in the blue boxes indicate self-efficacy, while the items in the red boxes indicate desire).

In the postsurvey (Figure 3), group 1 remained similar to group 1 in the presurvey (high–high), but the percentage of students in the group decreased to 67%. The other two groups, however, changed latent patterns over the course of the semester. In the postsurvey, group 2 included students with slightly high desires to pursue careers in STEM fields, compared to moderate desires reported in the presurvey, and low self-belief (**high-low, HL**), representing 17% of the population. The trends in postgroup 2 compared to pregroup 2 are similar; however, there was a slight increase in the probability plot values for the postsurvey relative to the presurvey. Group 3 comprised students with low desires and low self-belief (**low-low, LL**), accounting for 16% of the population. Students in the high-desire, high-efficacy (HH) group were characterized by agreeing with all 9 of the 9 mATSI indicators. For example, there was a 99% likelihood that these students look forward to taking a science class (compared to 100% in the presurvey), 90% likelihood they believe science is easy for them (compared to 93% in the presurvey), and a 95% likelihood that they believed they would do well in science classes (compared to 97% in the presurvey). Students in the high-desire, low-efficacy group (HL) were characterized by agreement (above 75% likelihood) with items that had to do with their desires to take science classes and disagreement (less than 25% likelihood) with items that indicated a belief in their abilities to do well in science classes. For example, there was a 93% likelihood that students in this group looked forward to taking a science class (compared to 84% in the presurvey), 20% likelihood they believe science is easy for them (compared to 7% in the presurvey), and a 25% likelihood that these students believed they would do well in science classes (compared to 0% in the presurvey). Students in the low-desire, low-efficacy (LL) group were characterized by disagreement (less than 25% likelihood) with most of the 9 mATSI items. For example, there was a 19% likelihood that students in this group looked forward to taking a science class, 9% likelihood they believe science is easy for them (compared to 41% in the presurvey), and a 21% likelihood that these students believed they would do well in science classes.

### Research Question 2: How Do Students Transition between the Identified Groups, and What Are the Characteristics of the Students That Make up the Groups?

To address research question 2 (RQ-2), we used the LTA transition matrix and explored the characteristics of the students who transitioned into the different groups. We also analyzed the questions from the postsurvey that addressed students’ perceptions after taking CHEM 1. From the 6 total groups (3 groups for the presurvey and 3 for the postsurvey), nine (9) possible transitions were estimated (Table 2). The LTA model transition matrix (Table 2) gives the probability of transitions into the 9 groups that have been identified.

**Table 2:** Invariant LTA Model Transition Matrix Showing the Probabilities of Group Transitions Based on Attitudes toward Science before and after Taking a General Chemistry Course

	Post-Attitude		
Pre-Attitude	*High–High*	*High-Low*	*Low-Low*
*High–High*	**0.869**	0.083	0.048
*Moderate-Low*	0.283	**0.549**	0.167
*Moderate-Moderate*	0.044	0.032	**0.924**

Looking at the transition table, the top-left cell (High–High to High–High transition) indicates that there is an 87% likelihood that students who began CHEM 1 with high desires and high self-belief will retain high desires and high self-belief at the end of the CHEM 1 semester (Table 2). This suggests that students who come into CHEM 1 with strong positive desires and self-belief in their abilities to do well are more likely to leave the course with these positive desires and self-belief.

Conversely, among students who entered CHEM 1 with moderate desires and moderate self-beliefs, there is a 92% likelihood that these students will leave CHEM 1with low desires and low self-belief (bottom-left cell) and very low likelihoods that they will leave with high desires and high self-belief (4%) or with high desires and low self-belief (3%) (Table 2). This trend suggests that students who come into CHEM 1 ambivalent about their desires to pursue science and their self-belief in their abilities to do well are more likely to leave CHEM 1 with low desires and self-belief. There are obviously other factors that could influence students’ desires and self-belief, but this trend suggests building the level of confidence of students prior to (or even during) taking CHEM 1 could influence their desires to pursue science and self-belief in their abilities.

Finally, students who entered CHEM 1 with moderate desires and low self-worth showed more varied transitions. Specifically, there is a 55% likelihood that these students will end the semester with high desires and low self-belief, which means that students in this group are almost 50% likely to transition into a different group. Our model showed a 28% likelihood to transition to high desires and high self-belief, and a 17% likelihood to transition to low desires and low self-belief (Table 2; middle row). This trend suggests that students in this group may be more impressionable in a general chemistry course, and further investigations into these students’ experiences in the classroom would be valuable in exploring how to increase the persistence of students in STEM fields.

Our findings suggest that a student’s perception of a course/subject matter when they enter a course can influence their desire and self-belief in their abilities, and this can ultimately influence retention in STEM fields.^53^ It may be beneficial to spend time and resources working on students’ self-belief (and not just cognitive skills, e.g., math preparation) before they start the course. Studies investigating the effect of these types of precourse interventions would provide more insight into the trends observed.

#### Characteristics of Students in Different Transition Groups

**Figure 4:**
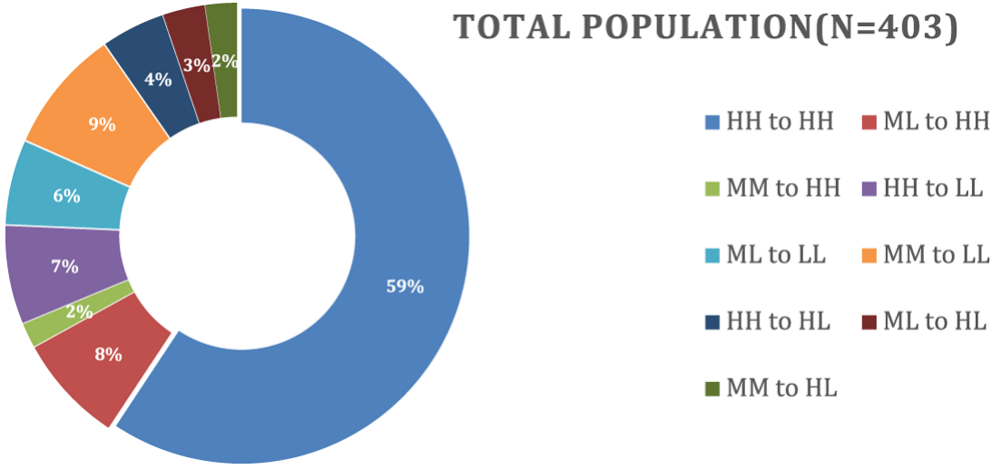
Nine different transition groups, total number of students in each group, and percentage of the total population.

Table 3 shows the different transition groups, the total number of individuals in each group, and the average grades. Most students (n = 239, 59% of the total population, 403) come into CHEM 1 with high desires and self-belief and leave CHEM 1 the same way. Furthermore, about 70% of the total population (n = 403) left CHEM 1 with high desires and self-belief. However, the remaining 30% are groups of students whose transitions and experiences in CHEM 1 are important but may often be overlooked because they represent a very small subset of the total population. Figure 4 presents a pictorial representation of Table 3.

**Table 3:** Nine Different Transition Groups, Total Number of Students in Each Group, and Percentage of the Total Population

	**n**	**% of the total population**
**Transition to High desires and high efficacy**		
High-desire and efficacy (Pre) to High-desire and efficacy (Post)	239	59.3%
Moderate-desire and low-efficacy (Pre) to High-desire and efficacy (Post)	31	7.7%
Moderate-desire and efficacy (Pre) to High-desire and efficacy (Post)	7	1.7%
**Transitions to Low desires and low efficacy**		
*High-desire and efficacy (Pre) to Low-desire and efficacy (Post)*	28	6.9%
*Moderate-desire and low-efficacy (Pre) to Low-desire and efficacy (Post)*	24	6.0%
*Moderate-desire and efficacy (Pre) to Low-desire and efficacy (Post)*	35	8.7%
**Transition to High Desires and low efficacy**		
*High-desire and efficacy (Pre)to High desire and low-efficacy (Post)*	18	4.5%
*Moderate-desire and low-efficacy (Pre)to High desire and low-efficacy (Post)*	12	3.0%
*Moderate-desire and efficacy (Pre)to High desire and low-efficacy (Post)*	9	2.2%

**Figure 5:**
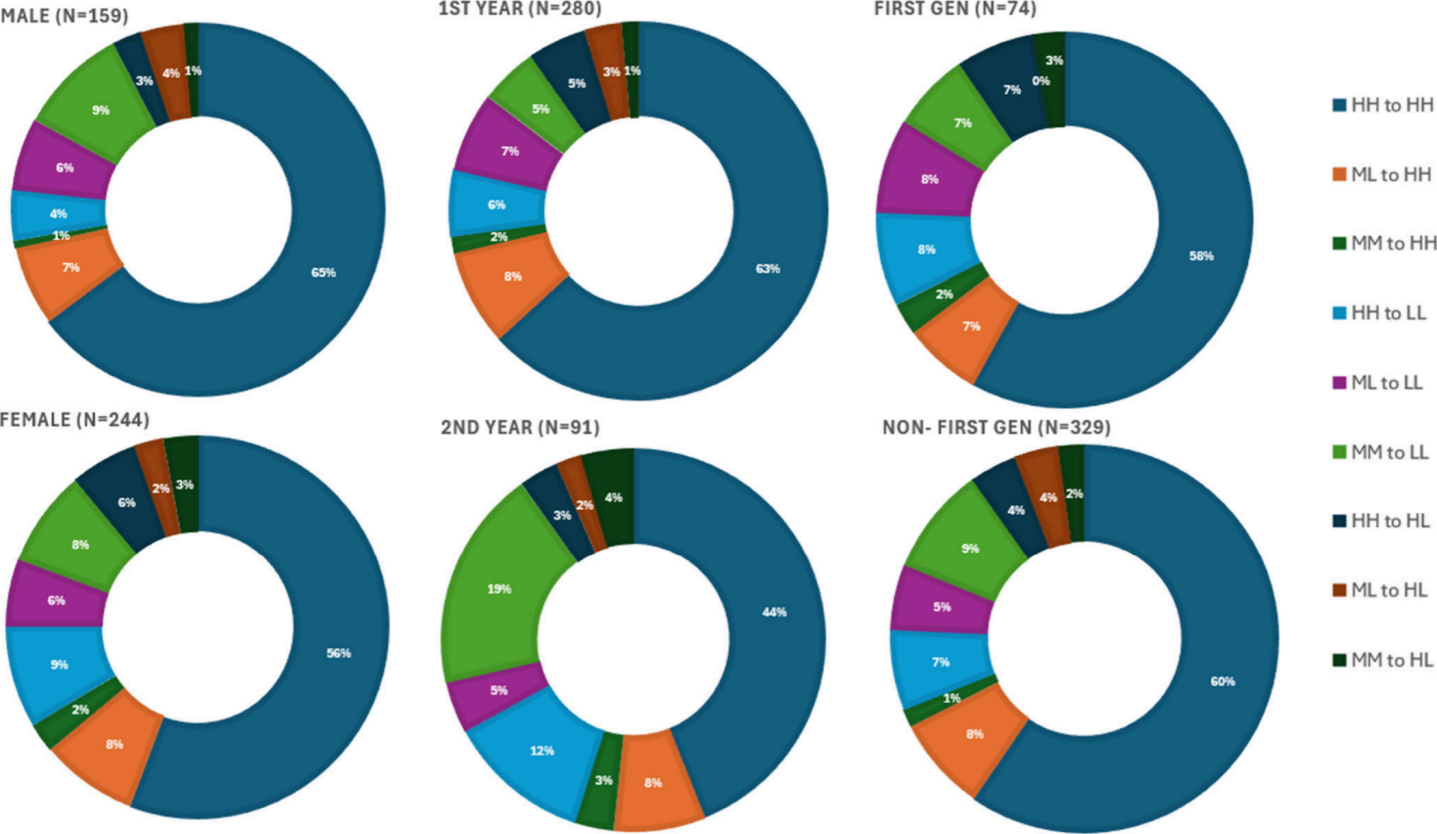
Breakdown of the percentages of the population that fell into the nine different transition groups.

To determine the characteristics of students in each transition class (e.g., HH to HH, ML to LL, etc.), we looked at each demographic group (i.e., male, female, first year, second year, first generation, and non-first-generation) and determined the distribution of these characteristics within each class (Figure 5). We did not find significant differences between first-generation and non-first-generation students, so the data from these groups are not discussed in detail. For this discussion, we determined over- or under-representation as any percentage greater or less than 1% of the total population percentage shown in Figure 4 (i.e., if the total population was 4%, over-representation would be 6% or more, while under-representation would be 2% or less).

#### Transitions into High Desire and High Self-Belief Groups (HH to HH, ML to HH, and MM to HH)

Male students and first-year students were overrepresented in the HH to HH group, meaning that students who identify as male or first-year students are more likely to come into CHEM 1 with high desires and self-belief and leave with high desires and self-belief. Conversely, female and second-year students were underrepresented in the HH to HH group, meaning that students who identify as female or second-year students are less likely to come into CHEM 1 with high desires and self-belief and leave the same way. The percentages for the other 2 transitions (ML to HH and MM to HH) were similar across all identity groups.

#### Transitions into Low Desire and Low Self-Belief Groups (HH to LL, ML to LL and MM to LL)

Female and second-year students were overrepresented in the MM to LL group, while male students were underrepresented. This means that students who identify as female or second-year students are more likely to come into CHEM 1 with moderate/midlevel desires and self-belief in their abilities to do well in science fields and leave with low desires and low self-belief in their abilities, while male students were less likely to do so. The HH to LL transition percentages were similar across the four identity groups. However, in the ML to LL transition group, second-year students were overrepresented, meaning that second year students were more likely to come into CHEM 1 with moderate desires and low self-belief in their abilities and leave with low desires and low self-belief. This is important to note because at the institution where the data were collected (and likely across other institutions), second year students are typically students who are retaking CHEM 1 for various reasons, including failure of the course the first time they took it.

#### Transitions into High Desire and Low Self-Belief Groups (HH to HL, ML to HL, and MM to HL)

Considering the sample sizes for these transition groups were considered, the percentages for these groups were similar. Female students and first-generation students were slightly overrepresented in the HH to HL group, first-generation students were not represented in the ML to HL group, and second-year students were slightly overrepresented in the MM to HL group. Studies that further investigate this group of students, particularly exploring why these students still have a high desire to pursue careers in science but low self-esteem, would be interesting.

Based on the data collected, male and first-year students are more likely to leave CHEM 1 with high desires and self-belief in their abilities, while female and second-year students are more likely to leave CHEM with low desires and self-belief. This is an interesting finding because research has shown that attrition in STEM fields happens in the first two years of college,^54^ and females are more likely to leave STEM fields than their male counterparts.^55^ If we can determine why specific groups of students are leaving with lower desires and self-belief and why other groups are leaving with higher desires and self-belief, we can hopefully understand better what influences the affective (noncognitive) domain for different student groups and potentially improve the retention rates of students across different identity groups. Dweck’s achievement motivation theory emphasizes students’ academic success also depends on noncognitive factors like attitudes, mindset, beliefs, and values; describing students’ mindsets or desires as malleable and tending to change over time.^56,57^ This suggests that the affective domain plays a major role in the retention and persistence of students in STEM fields and that developing programs focused on improving the affective domain instead of just the cognitive domain may be necessary in the quest for diversifying STEM fields.

### Research Question 3: What Relationship Exists between Students’ Perceptions and Their Final Course Grades?

To address the last research question (RQ −3), we compared student grades to their latent transition membership (Table 4). The final grades were obtained from the registrar’s office and were converted to numbers based on the 4.0 GPA scale (A = 4, B = 3, etc.) and the average grades for the members of each group was reported. For example, the average grade of students in the HH to HH group was 3.16 out of 4 (∼80%), which means the average student earned about 80% in the course. The results suggest a positive relationship between students’ final course grades and group membership (Table 4). In general, students who transitioned into the high desire and high self-belief (HH to HH, ML to HH, and MM to HH) group had higher grades, while students who transitioned into the low desire and low self-belief (HH to LL, ML to LL, and MM to LL) group had lower grades. In general, male students, first-year students, and non first-generation students tended to have higher grades compared to the other student groups. This is an interesting trend that suggests grades may not be the only factor that plays a role in students’ desire and self-efficacy toward chemistry courses.

**Table 4:** Relationship between Transition Groups and Final Course Grades

	**HH to HH** (*n* = 239)	**ML to HH** (*n* = 31)	**MM to HH** (*n* = 7)	**HH to LL** (*n* = 28)	**ML to LL** (*n* = 24)	**MM to LL** (*n* = 35)	**HH to HL** (*n* = 18)	**ML to HL** (*n* = 12)	**MM to HL** (*n* = 9)
**Average grade**	3.16	3.12	2.97	2.17	2.58	2.19	2.82	3.00	2.58
**Male**	3.23	2.90	3.20	2.54	2.48	2.34	2.80	3.34	2.00
**Female**	3.10	3.24	2.94	2.05	2.65	2.08	2.83	2.66	2.74
**First year**	3.34	3.30	3.00	2.23	2.66	2.46	3.03	3.20	3.00
**Second year**	2.62	2.40	2.94	2.05	2.20	1.98	1.86	2.40	2.40
**First Gen**	3.03	3.04	3.20	2.08	2.40	2.24	2.00	N/A	2.40
**Non-FG**	3.18	3.14	2.88	2.19	2.63	2.18	3.08	3.00	2.63

For the high desire and low self-belief transitions (HH to HL, ML to HL, and MM to HL), these students seem to have higher course grades than students in the low desire and low self-belief groups but lower grades than students in the high desire and self-belief groups. This trend suggests that this group of students who transition into the HL class may have other factors that influence their desire and self-belief in their abilities in addition to their grades in the course.

As part of the postsurvey, we asked students to select factors influencing their attitudes toward science (Supporting Information). Students were allowed to select all factors they believed applied to them from a list of factors (course instructor, lab instructor, other science courses and instructors, and course structure). We analyzed the data by combining transition groups based on the group they transitioned into (e.g., HH to HH, ML to HH, and MM to HH were all grouped together and labeled HH). When we looked at the self-reported data that asked for factors that influenced students’ perceptions of science (Table 5), the results showed that the majority of students who transitioned into HH (59%) or LL (63%) groups believed the course instructor influenced their views toward science, compared to 45% of students who transitioned into HL. Furthermore, a higher percentage of students who transitioned into HL (32%) believed the course structure influenced their perceptions, compared to 23% and 28% for the HH and LL groups, respectively. Studies further investigating what “course structure” means to students and how they believe it influences their perceptions would be beneficial. Interestingly, compared to students in the HH (13%) and LL (12%) groups, very few students in the HL group (3%) believed the lab instructor (who is different from the course instructor and is typically a graduate student) influenced their perceptions. Further qualitative studies investigating why and how these different groups of students believe certain factors influence their perceptions could benefit researchers, practitioners, and students.

**Table 5:** Factors That Influenced Students’ Perception of Science in CHEM 1

**Factors**	**HH** (*n* = 274)	**LL** (*n* = 82)	**HL** (*n* = 38)
*Course Instructor*	59%	63%	45%
*Course Structure (including course content and exams)*	23%	28%	32%
*Lab instructor*	13%	12%	3%
*Other Course/Instructor*	14%	11%	13%
*Stayed the same*	20%	10%	21%

### Limitations

There are several limitations to the study. Even though the total population was large enough for LCA (403), some of the transition groups had very small sample sizes, which can be problematic for latent class analysis.^27^ In addition, some of the identity groups had small sample sizes, which could also be problematic for statistical analysis and conclusions drawn. We also recognize that other factors could have influenced student attitudes over the course of the semester, but this study does not explore these. Another limitation could have been how we categorized the responses (grouping *strongly agree* and *agree*, and *strongly disagree* and *disagree*, and ignoring *neither agree nor disagree* responses). Categorizing in this way could have missed some patterns that could limit the findings in the study.

## Conclusion and Implications

Mixture modeling allows researchers to take a person-centered approach with quantitative data, uncovering trends that traditional quantitative analysis may miss. This paper seeks to highlight LCA and LTA as alternative methods researchers can use to explore nuances in data, as we work toward exploring the experiences of different student groups in STEM fields and supporting them in their quest to be successful in STEM fields. Using mixture modeling analyses, we found that even though the majority of the population in this study left CHEM 1 with high desires to pursue science and high self-belief in their abilities to be successful in subsequent science courses and a small group of students left with low desires and low self-efficacy (which is expected), another group emerged: a small subset of students left CHEM 1 with high desires, but low self-belief in their abilities. Based on this finding, further studies investigating the lived experiences of students who leave CHEM with high desires and low self-efficacy can help both researchers and instructors implement practices that can help support these students and improve their persistence in STEM fields. With these complementary approaches, educators and researchers can gain more defined ways of understanding their students, especially those that could appear insignificant, allowing educators to develop more tailored teaching strategies that accommodate the diverse needs of those learners. This is especially important as we (the larger discipline-based education research community) seek to continue to increase the representation of student groups that are typically underrepresented in STEM fields (e.g., underrepresented racial and ethnic groups, gender groups, groups with low socioeconomic status, and first-generation students).

Furthermore, the authors recommend that instructors be more intentional about providing tools to their students early in the semester that can help improve their perceptions of science and STEM fields. For example, highlighting scientists from underrepresented groups during class could help improve the perceptions of students who identify with these groups. Instructors could also collect survey data halfway through the semester, asking students about their current perceptions of science/STEM and asking for ways they believe these perceptions could be improved. These tools and recommendations can be incorporated across other chemistry and STEM courses.

## Supporting Information Available

Presurvey items measuring students’ perceptions; postsurvey items measuring students’ perceptions; R-codes to run LCA and LTA analysis (PDF, DOCX)
