# Supplementary material for: Investigating the Evolution of Student Attitudes toward Science in a General Chemistry Course Using Latent Class and Latent Transition Analysis
Source: J Chem Educ. 2025 Apr 22;102(5):1745–54. doi: 10.1021/acs.jchemed.4c01247 (PMC12080113; doi:10.1021/acs.jchemed.4c01247)
Supplement: Supplementary file 1 — ed4c01247_si_001.pdf [file ed4c01247_si_001.pdf]

Investigating the Evolution of Student Attitudes Towards Science in a General Chemistry Course Using Latent Class and Latent Transition Analysis

Oluwatobi O. Odeleye*[1], Oluwaseun D. Agunbiade[1], Adam Garber[2] and Karen Nylund-Gibson[2]

[1]Department of Chemistry, West Virginia University, Morgantown, WV, USA 26505

[2]University of California – Santa Barbara, Santa Barbara, CA, USA 93106

Supporting Information

The following documents are included:

- Pre-survey measuring students' perceptions
- Post-survey measuring students' perceptions
- R-codes to conduct the LCA and LTA analyses

Pre_Survey_Perceptions_STEM_FA_2022

Consent to Participate in Research at West Virginia University

IRB Protocol #: 207611431

Department of Chemistry
7/2022

Dear Prospective Participant,
This letter is a request for you to participate in a research project geared towards determining students' perceptions of science fields. This project is being conducted by Oluwatobi Odeleye, PhD, in the Department of Chemistry at WVU.
If you decide to participate, you will be asked to complete the survey that follows, which asks about your perceptions of science fields. Your participation in this project will take approximately 5 -10 minutes. You must be 18 years of age or older to participate. You may receive extra credit at the discretion of your instructor for participating. Your participation in this project will be kept confidential, and your instructors will not access your responses until final grades have been submitted. Your participation is entirely voluntary. You may skip any question that you do not wish to answer, and you may discontinue at any time. As a student, your class standing, grades, student work status, or status on an athletic team, if applicable, will not be affected if you decide not to participate or withdraw.
The West Virginia University Institutional Review Board's review of this research project is on file with the WVU Office of Human Research Protections. If you have any questions about this research project, please feel free to contact me at oluwatobi.odeleye@mail.wvu.edu. If I am your instructor, then please contact Nghiem Tieu (nut00001@mix.wvu.edu)or the WVU Office of Human Research Protections at 304-293-7073. I hope that you will participate in this research project, as it could help us better understand your perceptions of science and the factors that influence them. This in turn can help us improve our general chemistry program here at WVU.
Thank you for your time and consideration. Sincerely,Oluwatobi O. Odeleye
By answering the survey questions, I agree to participate in this research and provide a response to all questions asked.. Please print this page for your records. Please select one of the choices below.

- ○ I agree to participate
- ○ I do not agree to participate

Q13 What is your mixID (e.g. xxx00005)? This is for your extra credit, so please enter this correctly.

____

Q26 Please select your instructor:

- ○ Prof. Odeleye
- ○ Prof. Schraf
- ○ Prof. Ely
- ○ Prof. Tinsley
- ○ Prof. Ratcliff

Q15 What is your major?

- ○ Pre-Professional (e.g. pre-med, pre-vet, etc.)
- ○ Engineering
- ○ Physical Science (e.g. chemistry, physics, mathematics, geology, astronomy)
- ○ Biological Science (including Biology)
- ○ Allied Health (e.g. nursing, PA, etc.)
- ○ Education
- ○ Other ____

Q43 What does the term "science" mean to you?

____

Q40 For the following statements, select what best describes your feeling about the field of science.
Survey questions based on Modified Attitudes Towards Science Inventory

Q27 Science is useful in helping to solve the problems of everyday life

- Strongly agree
- Agree
- Somewhat agree
- Neither agree nor disagree
- Somewhat disagree
- Disagree
- Strongly disagree

Q31 Science is helpful in understanding today's world

- Strongly agree
- Agree
- Somewhat agree
- Neither agree nor disagree
- Somewhat disagree
- Disagree
- Strongly disagree

Q35 Science is of great importance to a country's development.

- Strongly agree
- Agree
- Somewhat agree
- Neither agree nor disagree
- Somewhat disagree
- Disagree
- Strongly disagree

Q45 Most people should study some science

- Strongly agree
- Agree
- Somewhat agree
- Neither agree nor disagree
- Somewhat disagree
- Disagree
- Strongly disagree

Q46 It is important to know science in order to get a good job

- Strongly agree
- Agree
- Somewhat agree
- Neither agree nor disagree
- Somewhat disagree
- Disagree
- Strongly disagree

Q28 Science is something I enjoy very much

- Strongly agree
- Agree
- Somewhat agree
- Neither agree nor disagree
- Somewhat disagree
- Disagree
- Strongly disagree

Q47 I enjoy reading science content that has not been assigned to me, or is not a part of a course I am taking.

- Strongly agree
- Agree
- Somewhat agree
- Neither agree nor disagree
- Somewhat disagree
- Disagree
- Strongly disagree

Q52 Please select "somewhat disagree"

- Strongly agree
- Agree
- Somewhat agree
- Neither agree nor disagree
- Somewhat disagree
- Disagree
- Strongly disagree

Q37 I look forward to taking science classes

- Strongly agree
- Agree
- Somewhat agree
- Neither agree nor disagree
- Somewhat disagree
- Disagree

- ◯ Strongly disagree

Q48 I like the challenge of science assignments

- ◯ Strongly agree
- ◯ Agree
- ◯ Somewhat agree
- ◯ Neither agree nor disagree
- ◯ Somewhat disagree
- ◯ Disagree
- ◯ Strongly disagree

Q41 I have a real desire to learn science

- ◯ Strongly agree
- ◯ Agree
- ◯ Somewhat agree
- ◯ Neither agree nor disagree
- ◯ Somewhat disagree
- ◯ Disagree
- ◯ Strongly disagree

Q49 It is important to me to understand the work I do in my science classes

- ◯ Strongly agree
- ◯ Agree
- ◯ Somewhat agree
- ◯ Neither agree nor disagree
- ◯ Somewhat disagree

- Disagree
- Strongly disagree

Q29 When I hear the word "science", I have a feeling of dislike.

- Strongly agree
- Agree
- Somewhat agree
- Neither agree nor disagree
- Somewhat disagree
- Disagree
- Strongly disagree

Q53 Please select "neither agree nor disagree"

- Strongly agree
- Agree
- Somewhat agree
- Neither agree nor disagree
- Somewhat disagree
- Disagree
- Strongly disagree

Q34 I feel tense when someone talks to me about science

- Strongly agree
- Agree
- Somewhat agree
- Neither agree nor disagree

- Somewhat disagree
- Disagree
- Strongly disagree

Q38 It makes me nervous to even think about doing science

- Strongly agree
- Agree
- Somewhat agree
- Neither agree nor disagree
- Somewhat disagree
- Disagree
- Strongly disagree

Q39 It scares me to have to take a science class

- Strongly agree
- Agree
- Somewhat agree
- Neither agree nor disagree
- Somewhat disagree
- Disagree
- Strongly disagree

Q36 I have a good feeling towards science classes

- Strongly agree
- Agree
- Somewhat agree

- Neither agree nor disagree
- Somewhat disagree
- Disagree
- Strongly disagree

Q30 Science is easy for me

- Strongly agree
- Agree
- Somewhat agree
- Neither agree nor disagree
- Somewhat disagree
- Disagree
- Strongly disagree

Q42 I do not do very well in science classes

- Strongly agree
- Agree
- Somewhat agree
- Neither agree nor disagree
- Somewhat disagree
- Disagree
- Strongly disagree

Q50 I usually understand what we are talking about in my science classes

- Strongly agree
- Agree

- Somewhat agree
- Neither agree nor disagree
- Somewhat disagree
- Disagree
- Strongly disagree

Q32 No matter how hard I try, I cannot understand science

- Strongly agree
- Agree
- Somewhat agree
- Neither agree nor disagree
- Somewhat disagree
- Disagree
- Strongly disagree

Q51 I often think, "I cannot do this", when a science assignment seems hard.

- Strongly agree
- Agree
- Somewhat agree
- Neither agree nor disagree
- Somewhat disagree
- Disagree
- Strongly disagree

Q54 Science teachers make science courses interesting

- Strongly agree

- Agree
- Somewhat agree
- Neither agree nor disagree
- Somewhat disagree
- Disagree
- Strongly disagree

Q55 Science teachers typically present the course material in a clear and understandable way

- Strongly agree
- Agree
- Somewhat agree
- Neither agree nor disagree
- Somewhat disagree
- Disagree
- Strongly disagree

Q56 Science teachers are willing to give their students individual help

- Strongly agree
- Agree
- Somewhat agree
- Neither agree nor disagree
- Somewhat disagree
- Disagree
- Strongly disagree

Q9 What is your gender?

- Female
- Male
- Other ____

Q25 What is your ethnicity?

- Asian
- Black/African
- Caucasian
- Hispanic/Latinx
- Native American
- Pacific Islander
- Mixed Race
- Prefer not to answer

Q14 What is your year in school?

- Freshman
- Sophomore
- Junior
- Senior
- Other

Post_Survey_Perceptions_STEM_FA_2022

Consent to Participate in Research at West Virginia University

IRB Protocol #: **207611431**

Department of Chemistry

7/2022

Dear Prospective Participant,

This letter is a request for you to participate in a research project geared towards determining students' perceptions of science fields. This project is being conducted by Oluwatobi Odeleye, PhD, in the Department of Chemistry at WVU.

If you decide to participate, you will be asked to complete the survey that follows, which asks

about your perceptions of science fields. Your participation in this project will take approximately 5 -10 minutes. You must be 18 years of age or older to participate. You may receive extra credit points at the discretion of your instructor. Your participation in this project will be kept confidential, and your instructors will not access your responses until final grades have been submitted. Your participation is entirely voluntary. You may skip any question that you do not wish to answer, and you may discontinue at any time. As a student, your class standing, grades, student work status, or status on an athletic team, if applicable, will not be affected if you decide not to participate or withdraw.
The West Virginia University Institutional Review Board's review of this research project is on file with the WVU Office of Human Research Protections. If you have any questions about this research project, please feel free to contact me at oluwatobi.odeleye@mail.wvu.edu. If I am your instructor, then please contact Nghiem Tieu (nut00001@mix.wvu.edu)or the WVU Office of Human Research Protections at 304-293-7073. I hope that you will participate in this research project, as it could help us better understand your perceptions of science and the factors that influence them. This in turn can help us improve our general chemistry program here at WVU. Thank you for your time and consideration. Sincerely,Oluwatobi O. Odeleye
By answering the survey questions, I agree to participate in this research and provide a response to all questions asked.. Please print this page for your records. Please select one of the choices below.

- ◯ I agree to participate
- ◯ I do not agree to participate

End of Block: Block 4

Start of Block: Default Question Block

Q13 What is your mixID number (e.g. xxx00005)? This is for your extra credit, so please enter this correctly.

____

Q26 Please select your instructor:

- ◯ Prof. Odeleye
- ◯ Prof. Schraf
- ◯ Prof. Ely
- ◯ Prof. Tinsley
- ◯ Prof. Ratcliff

Q27 How has your perception of ***science*** in general changed since you have been in your current chemistry class?

- ◯ It has not changed
- ◯ It has become more positive
- ◯ It has become more negative

Q28 After taking your general chemistry course this semester, which of the following has influenced your perception of ***science*** this semester?

- ☐ The course instructor
- ☐ The lab TA/instructor
- ☐ Other science courses you are taking this semester
- ☐ Other science instructors
- ☐ The course structure (including course content and exams)
- ☐ Other (please provide a specific answer) ____
- ☐ My perception has stayed the same.

Q29 How has your perception of ***chemistry*** changed since you have been in your current chemistry class?

- ○ It has not changed
- ○ It has become more positive
- ○ It has become more negative

Q30 After taking your general chemistry course this semester, which of the following has influenced your perception of ***chemistry*** this semester?

- ☐ The course instructor
- ☐ The lab TA/instructor
- ☐ Other science courses you are taking this semester
- ☐ Other science instructors
- ☐ The course structure (including the course content and exams)
- ☐ Other (please provide a specific answer) ____
- ☐ My perception has stayed the same.

Q31 As a result of taking this course...

- ◯ I believe I can do well in science courses
- ◯ I believe I cannot do well in science courses
- ◯ This course has not influenced my belief in myself

Q32 As a result of taking this course...

- ◯ I am not interested in pursuing a career in science
- ◯ I am interested in pursuing a career in science
- ◯ This course has not influenced my desire to pursue a career in science

Q34 As a result of taking this course...

- ◯ I believe I can do well in other chemistry courses
- ◯ I believe I cannot do well in other chemistry courses
- ◯ This course has not influenced my belief in myself

Q35 As a result of taking this course...

- ◯ I am not interested in pursuing a career in chemistry
- ◯ I am interested in pursuing a career in chemistry
- ◯ This course has not influenced my desire to pursue a career in chemistry

Q36 Are there any other factors you believe have influenced your perception of science fields? If yes, please explain below.

____

Q37 Are there any other factors you believe have influenced your perception of the field of chemistry? If yes, please explain below.

____

Q41 For the following statements, select what best describes your feeling about the field of science.
Survey questions based on Modified Attitudes Towards Science Inventory

Q14 What is your year in school?

- ◯ Freshman
- ◯ Sophomore
- ◯ Junior
- ◯ Senior
- ◯ Other

Q27 Science is useful in helping to solve the problems of everyday life

- ◯ Strongly agree
- ◯ Agree
- ◯ Somewhat agree
- ◯ Neither agree nor disagree
- ◯ Somewhat disagree
- ◯ Disagree
- ◯ Strongly disagree

Q31 Science is helpful in understanding today's world

- ◯ Strongly agree
- ◯ Agree
- ◯ Somewhat agree
- ◯ Neither agree nor disagree
- ◯ Somewhat disagree
- ◯ Disagree
- ◯ Strongly disagree

Q35 Science is of great importance to a country's development.

- Strongly agree
- Agree
- Somewhat agree
- Neither agree nor disagree
- Somewhat disagree
- Disagree
- Strongly disagree

Q45 Most people should study some science

- Strongly agree
- Agree
- Somewhat agree
- Neither agree nor disagree
- Somewhat disagree
- Disagree
- Strongly disagree

Q46 It is important to know science in order to get a good job

- Strongly agree
- Agree
- Somewhat agree
- Neither agree nor disagree
- Somewhat disagree
- Disagree
- Strongly disagree

Q28 Science is something I enjoy very much

- Strongly agree
- Agree
- Somewhat agree
- Neither agree nor disagree
- Somewhat disagree
- Disagree
- Strongly disagree

Q47 I enjoy reading science content that has not been assigned to me, or is not a part of a course I am taking.

- Strongly agree
- Agree
- Somewhat agree
- Neither agree nor disagree
- Somewhat disagree
- Disagree
- Strongly disagree

Q52 Please select "somewhat disagree"

- Strongly agree
- Agree
- Somewhat agree
- Neither agree nor disagree
- Somewhat disagree
- Disagree

- Strongly disagree

Q37 I look forward to taking science classes

- Strongly agree
- Agree
- Somewhat agree
- Neither agree nor disagree
- Somewhat disagree
- Disagree
- Strongly disagree

Q48 I like the challenge of science assignments

- Strongly agree
- Agree
- Somewhat agree
- Neither agree nor disagree
- Somewhat disagree
- Disagree
- Strongly disagree

Q41 I have a real desire to learn science

- Strongly agree
- Agree
- Somewhat agree
- Neither agree nor disagree
- Somewhat disagree

- Disagree
- Strongly disagree

Q49 It is important to me to understand the work I do in my science classes

- Strongly agree
- Agree
- Somewhat agree
- Neither agree nor disagree
- Somewhat disagree
- Disagree
- Strongly disagree

Q29 When I hear the word "science", I have a feeling of dislike.

- Strongly agree
- Agree
- Somewhat agree
- Neither agree nor disagree
- Somewhat disagree
- Disagree
- Strongly disagree

Q53 Please select "neither agree nor disagree"

- Strongly agree
- Agree
- Somewhat agree
- Neither agree nor disagree

- Somewhat disagree
- Disagree
- Strongly disagree

Q34 I feel tense when someone talks to me about science

- Strongly agree
- Agree
- Somewhat agree
- Neither agree nor disagree
- Somewhat disagree
- Disagree
- Strongly disagree

Q38 It makes me nervous to even think about doing science

- Strongly agree
- Agree
- Somewhat agree
- Neither agree nor disagree
- Somewhat disagree
- Disagree
- Strongly disagree

Q39 It scares me to have to take a science class

- Strongly agree
- Agree
- Somewhat agree

- Neither agree nor disagree
- Somewhat disagree
- Disagree
- Strongly disagree

Q36 I have a good feeling towards science classes

- Strongly agree
- Agree
- Somewhat agree
- Neither agree nor disagree
- Somewhat disagree
- Disagree
- Strongly disagree

Q30 Science is easy for me

- Strongly agree
- Agree
- Somewhat agree
- Neither agree nor disagree
- Somewhat disagree
- Disagree
- Strongly disagree

Q42 I do not do very well in science classes

- Strongly agree
- Agree

- Somewhat agree
- Neither agree nor disagree
- Somewhat disagree
- Disagree
- Strongly disagree

Q50 I usually understand what we are talking about in my science classes

- Strongly agree
- Agree
- Somewhat agree
- Neither agree nor disagree
- Somewhat disagree
- Disagree
- Strongly disagree

Q32 No matter how hard I try, I cannot understand science

- Strongly agree
- Agree
- Somewhat agree
- Neither agree nor disagree
- Somewhat disagree
- Disagree
- Strongly disagree

Q51 I often think, "I cannot do this", when a science assignment seems hard.

- Strongly agree

- Agree
- Somewhat agree
- Neither agree nor disagree
- Somewhat disagree
- Disagree
- Strongly disagree

Q54 Science teachers make science courses interesting

- Strongly agree
- Agree
- Somewhat agree
- Neither agree nor disagree
- Somewhat disagree
- Disagree
- Strongly disagree

Q55 Science teachers typically present the course material in a clear and understandable way

- Strongly agree
- Agree
- Somewhat agree
- Neither agree nor disagree
- Somewhat disagree
- Disagree
- Strongly disagree

Q56 Science teachers are willing to give their students individual help

- Strongly agree
- Agree
- Somewhat agree
- Neither agree nor disagree
- Somewhat disagree
- Disagree
- Strongly disagree

Q15 What is your major?

- Pre-Professional (e.g. pre-med, pre-vet, etc.)
- Engineering
- Physical Science (e.g. chemistry, physics, mathematics, geology, astronomy)
- Biological Science (including Biology)
- Allied Health (e.g. nursing, PA, etc.)
- Education
- Other ____

Q9 What is your gender?

- Female
- Male
- Other ____

Q25 What is your ethnicity?

- Asian
- Black/African
- Caucasian

- ○ Hispanic/Latinx
- ○ Native American
- ○ Pacific Islander
- ○ Mixed Race
- ○ Prefer not to answer

R codes for LCA analysis

(https://github.com/MM4DBER/Intro_to_LCA/blob/main/Intro_to_LCA.Rmd)

````
---
title: "Introduction to Latent Class Analysis (LCA) with MplusAutomation"
subtitle: "*Enumeration, Summarizing Results, Exploring Response Patterns*"
author: "MM4DEBER Training Team"
date: "Updated: `r format(Sys.time(), '%B %d, %Y')`"
output:
  html_document:
    theme: flatly
  pdf_document:
    toc: yes
editor_options:
  markdown:
    wrap: sentence
---

```{r setup, include=FALSE}
knitr::opts_chunk$set(echo = TRUE, warning = FALSE, message = FALSE,
tidy.opts=list(width.cutoff=60))
```

# ---------------------------------------------------------------------------------

![](figures/mm4dber_Logo.jpg){style="float: left;" width="300"}

![](figures/NSF-Logo.png){style="float: left;" width="300"}

# ---------------------------------------------------------------------------------

> Mixture Modeling for Discipline Based Education Researchers (MM4DBER) is an NSF funded
training grant to support STEM Education scholars in integrating mixture modeling into their
research.

#### Download project materials here: [GitHub
Repository](https://github.com/MM4DBER/Intro_to_LCA)
````

Follow along: [Video Tutorial](https://www.youtube.com/watch?v=fPpcScLZFRI)

Return to landing page here: [MM4DBER Landing Page](https://mm4dber.github.io/)

Example: Bullying in Schools

- To demonstrate mixture modeling in the training program and online resource components of the IES grant we utilize the *Civil Rights Data Collection (CRDC)* (CRDC) data repository.
- The CRDC is a federally mandated school-level data collection effort that occurs every other year.
- This public data is currently available for selected latent class indicators across 4 years (2011, 2013, 2015, 2017) and all US states.
- In this example, we use the Arizona state sample.
- We utilize six focal indicators which constitute the latent class model in our example; three variables which report on harassment/bullying in schools based on disability, race, or sex, and three variables on full-time equivalent school staff hires (counselor, psychologist, law enforcement).
- This data source also includes covariates on a variety of subjects and distal outcomes reported in 2018 such as math/reading assessments and graduation rates.

Load packages

```{r}

library(tidyverse)
library(haven)
library(glue)
library(MplusAutomation)
library(here)
library(janitor)
library(gt)
library(cowplot)
library(DiagrammeR)
library(webshot2)

```

Variable Description

```{r, echo=FALSE}
tribble(
  ~"Name",      ~"Label",  ~"Values",
#--------------|--------------------------------|-----|,
"leaid",   "District Identification Code", "",
"ncessch",   "School Identification Code", "",
"report_dis","Number of students harassed or bullied on the basis of disability","0 = No reported incidents, 1 = At least one reported incident",
```

```
"report_race","Number of students harassed or bullied on the basis of race, color, or national origin",  "0 = No reported incidents, 1 = At least one reported incident",
"report_sex", "Number of students harassed or bullied on the basis of sex","0 = No reported incidents, 1 = At least one reported incident",
"counselors_fte", "Number of full time equivalent counselors hired as school staff","0 = No staff present, 1 = At least one staff present",
"psych_fte", "Number of full time equivalent psychologists hired as school staff",  "0 = No staff present, 1 = At least one staff present",
"law_fte", "Number of full time equivalent law enforcement officers hired as school staff",  "0 = No staff present, 1 = At least one staff present") %>%
gt() %>%
  tab_header(
    title = "LCA indicators"  # Add a title
    ) %>%
  tab_options(
    table.width = pct(75)
  ) %>%
  tab_footnote(
    footnote = "Civil Rights Data Collection (CRDC)",
    location = cells_title())
```

Save table

```{r, eval=FALSE}
gtsave("figures/variables.png")
```

Variables have been transformed to be dichotomous indicators using the following coding strategy

- Harassment and bullying count variables are recoded `1` if the school reported at least one incident of harassment (`0` indicates no reported incidents).
- On the original scale reported by the CDRC staff variables for full time equivalent employees (FTE) are represented as `1` and part time employees are represented by values between `1` and `0`.
- Schools with greater than one staff of the designated type are represented by values greater than 1.
- All values greater than zero were recorded as `1` indicating that the school has a staff present on campus at least part time.
Schools with no staff of the designated type are indicated by `0` for the dichotomous variable.

```{r, echo=FALSE, eval=TRUE, fig.align='center'}

grViz(" digraph cfa_model {

# The `graph` statement - No editing needed
```

```
    graph [layout = dot, overlap = true]

# Two `node` statements

# One for measured variables (box)

    node [shape=box]
    report_dis report_race report_sex counselors_fte psych_fte law_fte;

# One for latent variables (circle)

    node [shape=circle]
    bully [label=<Bullying <br/>C<sub>k</sub>>];

# `edge` statements

    edge [minlen = 2]
    bully -> {report_dis report_race report_sex counselors_fte psych_fte law_fte}

 }")
```

Prepare Data
```{r, eval=TRUE}
df_bully <- read_csv(here("data", "crdc_lca_data.csv")) %>%
  clean_names() %>%
  select(report_dis, report_race, report_sex, counselors_fte, psych_fte, law_fte)
```

Descriptive Statistics
```{r}
# Set up data to find proportions of binary indicators
ds <- df_bully %>%
  pivot_longer(c(report_dis, report_race, report_sex, counselors_fte, psych_fte, law_fte),
               names_to = "variable")


# Create table of variables and counts, then find proportions and round to 3 decimal places
prop_df <- ds %>%
  count(variable, value) %>%
  group_by(variable) %>%
  mutate(prop = n / sum(n)) %>%
  ungroup() %>%
  mutate(prop = round(prop, 3))


# Make it a gt() table
```

````
prop_table <- prop_df %>%
  gt(groupname_col = "variable", rowname_col = "value") %>%
  tab_stubhead(label = md("*Values*")) %>%
  tab_header(
    md(
      "Variable Proportions"
    )
  ) %>%
  cols_label(
    variable = md("*Variable*"),
    value = md("*Value*"),
    n = md("*N*"),
    prop = md("*Proportion*")
  )

prop_table
```

```{r, eval=FALSE}
# Save in figures folder
gtsave(prop_table, here("figures", "prop_table.png"))
```

---------------------------------------------------------------------------

### A Quick Introduction to `MplusAutomation`

Below is a template for `mplusObject()` & `mplusModeler()` functions. Use this template to run
statistical models with Mplus.
```{r, eval = FALSE}

m_template  <- mplusObject(

  TITLE =
    "",

  VARIABLE =
    "",

  ANALYSIS =
    "",

  PLOT =
    "",

  OUTPUT =
    "",

  usevariables = colnames(),
  rdata =  )
````

```
m_template_fit <- mplusModeler(m_template,
                   dataout=here("", ".dat"),
                   modelout=here("", ".inp"),
                   check=TRUE, run = TRUE, hashfilename = FALSE)

```

Enumeration

This code uses the `mplusObject` function in the `MplusAutomation` package and saves all model runs in the `enum` folder.
```{r, eval=FALSE}

lca_6  <- lapply(1:6, function(k) {
  lca_enum  <- mplusObject(

    TITLE = glue("{k}-Class"),

    VARIABLE = glue(
    "categorical = report_dis-law_fte;
     usevar = report_dis-law_fte;
     classes = c({k}); "),

  ANALYSIS =
   "estimator = mlr;
    type = mixture;
    starts = 200 100;
    processors = 10;",

  OUTPUT = "sampstat residual tech11 tech14;",

  PLOT =
    "type = plot3;
    series = report_dis-law_fte(*);",

  usevariables = colnames(df_bully),
  rdata = df_bully)

lca_enum_fit <- mplusModeler(lca_enum,
                 dataout=glue(here("enum", "bully.dat")),
                 modelout=glue(here("enum", "c{k}_bully.inp")),
                 check=TRUE, run = TRUE, hashfilename = FALSE)
})

```

IMPORTANT: Before moving forward, make sure to open each output document to ensure models were estimated normally.

Table of Fit

First, extract data
```{r}

output_bully <- readModels(here("enum"), filefilter = "bully", quiet = TRUE)

enum_extract <- LatexSummaryTable(output_bully,
    keepCols = c("Title","Parameters","LL","BIC","aBIC",
    "BLRT_PValue","T11_VLMR_PValue","Observations"),
    sortBy = "Title")

allFit <- enum_extract %>%
  mutate(CAIC = -2 * LL + Parameters * (log(Observations) + 1)) %>%
  mutate(AWE = -2 * LL + 2 * Parameters * (log(Observations) + 1.5)) %>%
  mutate(SIC = -.5 * BIC) %>%
  mutate(expSIC = exp(SIC - max(SIC))) %>%
  mutate(BF = exp(SIC - lead(SIC))) %>%
  mutate(cmPk = expSIC / sum(expSIC)) %>%
  dplyr::select(1:5, 9:10, 6:7, 13, 14) %>%
  arrange(Parameters)
```

Then, create table
```{r}
fit_table1 <- allFit %>%
  gt() %>%
  tab_header(title = md("**Model Fit Summary Table**")) %>%
  cols_label(
    Title = "Classes",
    Parameters = md("Par"),
    LL = md("*LL*"),
    T11_VLMR_PValue = "VLMR",
    BLRT_PValue = "BLRT",
    BF = md("BF"),
    cmPk = md("*cmPk*")
  ) %>%
  tab_footnote(
    footnote = md(
      "*Note.* Par = Parameters; *LL* = model log likelihood;
BIC = Bayesian information criterion;
aBIC = sample size adjusted BIC; CAIC = consistent Akaike information criterion;
AWE = approximate weight of evidence criterion;
BLRT = bootstrapped likelihood ratio test p-value;
VLMR = Vuong-Lo-Mendell-Rubin adjusted likelihood ratio test p-value;
*cmPk* = approximate correct model probability."
    ),
locations = cells_title()
  ) %>%
  tab_options(column_labels.font.weight = "bold") %>%
```

```
  fmt_number(c(3:7),
             decimals = 2) %>%
  sub_missing(1:11,
              missing_text = "--") %>%
  fmt(c(8:9, 11),
  fns = function(x)
  ifelse(x < 0.001, "<.001", scales::number(x, accuracy = .01))
  ) %>%
  fmt(10,fns = function (x)
     ifelse(x > 100, ">100", scales::number(x, accuracy = .01))
  ) %>%
  tab_style(
    style = list(
      cell_text(weight = "bold")),
    locations = list(cells_body(
     columns = BIC,
     row = BIC == min(BIC[c(1:6)]) # Change this to the number of classes you are evaluating
    ),
    cells_body(
     columns = aBIC,
     row = aBIC == min(aBIC[1:6])
    ),
    cells_body(
     columns = CAIC,
     row = CAIC == min(CAIC[1:6])
    ),
    cells_body(
     columns = AWE,
     row = AWE == min(AWE[1:6])
    ),
    cells_body(
     columns = cmPk,
     row =  cmPk == max(cmPk[1:6])
     ),
    cells_body(
     columns = BF,
     row =  BF > 10),
    cells_body(
     columns =  T11_VLMR_PValue,
     row =  ifelse(T11_VLMR_PValue < .05 & lead(T11_VLMR_PValue) > .05,
T11_VLMR_PValue < .05, NA)),
    cells_body(
     columns =  BLRT_PValue,
     row =  ifelse(BLRT_PValue < .05 & lead(BLRT_PValue) > .05, BLRT_PValue < .05, NA))
  )
)

fit_table1
```

Save table

```{r, eval = FALSE}
gtsave(fit_table1, here("figures", "fit_table1.png"))
```

Information Criteria Plot
```{r height=5, width=7}
allFit %>%
  dplyr::select(2:7) %>%
  rowid_to_column() %>%
  pivot_longer(`BIC`:`AWE`,
               names_to = "Index",
               values_to = "ic_value") %>%
  mutate(Index = factor(Index,
                        levels = c ("AWE", "CAIC", "BIC", "aBIC"))) %>%
  ggplot(aes(
    x = rowid,
    y = ic_value,
    color = Index,
    shape = Index,
    group = Index,
    lty = Index
  )) +
  geom_point(size = 2.0) + geom_line(size = .8) +
  scale_x_continuous(breaks = 1:nrow(allFit)) +
  scale_colour_grey(end = .5) +
  theme_cowplot() +
  labs(x = "Number of Classes", y = "Information Criteria Value", title = "Information Criteria") +
  theme(
    text = element_text(family = "serif", size = 12),
    legend.text = element_text(family="serif", size=12),
    legend.key.width = unit(3, "line"),
    legend.title = element_blank(),
    legend.position = "top"
  )
```

Save figure
```{r, eval=FALSE}
ggsave(here("figures", "info_criteria.png"), dpi=300, height=5, width=7, units="in")
```

Compare Class Solutions

Compare probability plots for $K = 1:6$ class solutions
```{r}
model_results <- data.frame()
```

````
for (i in 1:length(output_bully)) {

  temp <- output_bully[[i]]$parameters$probability.scale %>%
    mutate(model = paste(i,"-Class Model"))

  model_results <- rbind(model_results, temp)
}

rm(temp)

compare_plot <-
  model_results %>%
  filter(category == 2) %>%
  dplyr::select(est, model, LatentClass, param) %>%
  mutate(param = as.factor(str_to_lower(param)))

compare_plot$param <- fct_inorder(compare_plot$param)

ggplot(
  compare_plot,
  aes(
    x = param,
    y = est,
    color = LatentClass,
    shape = LatentClass,
    group = LatentClass,
    lty = LatentClass
  )
) +
  geom_point() +
  geom_line() +
  scale_colour_viridis_d() +
  facet_wrap( ~ model, ncol = 2) +
  labs(title = "Bullying Items",
       x = " ", y = "Probability") +
  theme_minimal() +
  theme(panel.grid.major.y = element_blank(),
                          axis.text.x = element_text(angle = -45, hjust = -.1))
```
````

Save figure

````
```{r, eval = FALSE}
ggsave(here("figures", "compare_kclass_plot.png"), dpi=300, height=5, width=7, units="in")
```
````

3-Class Probability Plot

Use the `plot_lca` function provided in the folder to plot the item probability plot. Details for this function are written in the document `plot_lca.txt`
````

```{r fig.height=6, fig.width=10}
source("plot_lca.txt")

plot_lca(model_name = output_bully$c3_bully.out)
```

Save figure
```{r, eval = FALSE}
ggsave(here("figures", "C3_bully_LCA_Plot.png"), dpi=300, height=5, width=7, units="in")
```

Observed Response Patterns

Save response frequencies for the 3-class model from the previous lab with `response is _____.dat` under `SAVEDATA.`
```{r, eval=FALSE}

patterns  <- mplusObject(

  TITLE = "C3 LCA - Save response patterns",

  VARIABLE =
  "categorical = report_dis-law_fte;
   usevar =  report_dis-law_fte;
   classes = c(3);",

  ANALYSIS =
   "estimator = mlr;
    type = mixture;
    starts = 0;
    processors = 10;
    optseed = 802779;",

  SAVEDATA =
   "File=savedata.dat;
    Save=cprob;
    response is resp_patterns.dat;
   !!! Code to save response frequency data !!!",

  OUTPUT = "residual patterns tech11 tech14",

  usevariables = colnames(df_bully),
  rdata = df_bully)

patterns_fit <- mplusModeler(patterns,
                    dataout=here("mplus", "bully.dat"),
                    modelout=here("mplus", "patterns.inp") ,
                    check=TRUE, run = TRUE, hashfilename = FALSE)
```

```

Note: You may see an error that says `<simpleError in bivarFitData[mPos, ] <- c(vars, values): number of items to replace is not a multiple of replacement length>`, the developers are aware of this and are working to fix it.

---------------------------------------------------------------------

Read in observed response pattern data and relabel the columns
```{r}
# Read in response frequency data that we just created:
patterns <- read_table(here("mplus", "resp_patterns.dat"),
                       col_names=FALSE, na = "*")

# Extract the column names
names <- names(readModels(here("mplus", "patterns.out"))[['savedata']])

# Add the names back to the dataset
colnames(patterns) <- c("Frequency", names)
```

Create a table with the top 5 unconditional response pattern, then top of conditional response pattern for each modal class assignment
```{r,eval=TRUE}
# Order responses by highest frequency
order_highest <- patterns %>%
  arrange(desc(Frequency))

# Loop `patterns` data to list top 5 conditional response patterns for each class
loop_cond  <- lapply(1:max(patterns$C), function(k) {
order_cond <- patterns %>%
  filter(C == k) %>%
  arrange(desc(Frequency)) %>%
  head(5)
  })

# Convert loop into data frame
table_data <- as.data.frame(bind_rows(loop_cond))

# Combine unconditional and conditional responses patterns
response_patterns <-  rbind(order_highest[1:5,], table_data)
```

Finally, use `{gt}` to make a nicely formatted table
```{r}
resp_table <- response_patterns %>%
  gt() %>%
    tab_header(
    title = "Observed Response Patterns",
    subtitle = html("Response patterns, estimated frequencies, estimated posterior class
probabilities and modal assignments")) %>%

````
    tab_source_note(
    source_note = md("Data Source: **Civil Rights Data Collection (CRDC)**")) %>%
    cols_label(
      Frequency = html("<i>f</i>_r"),
    REPORT_D = "Harrassment: Disability",
    REPORT_R = "Harrassment: Race",
    REPORT_S = "Harrassment: Sex",
    COUNSELO = "Staff: Counselor",
    PSYCH_FT = "Staff: Psychologist",
    LAW_FTE = "Staff: Law Enforcement",
    CPROB1 = html("P_{<i>k</i>}=1"),
    CPROB2 = html("P_{<i>k</i>}=2"),
    CPROB3 = html("P_{<i>k</i>}=3"),
    C = md("*k*")) %>%
  tab_row_group(
    label = "Unconditional response patterns",
    rows = 1:5) %>%
  tab_row_group(
    label = md("*k* = 1 Conditional response patterns"),
    rows = 6:10) %>% #EDIT THESE VALUES BASED ON THE LAST COLUMN
  tab_row_group(
    label = md("*k* = 2 Conditional response patterns"),
    rows = 11:15) %>% #EDIT THESE VALUES BASED ON THE LAST COLUMN
  tab_row_group(
    label = md("*k* = 3 Conditional response patterns"),
    rows = 16:20) %>% #EDIT THESE VALUES BASED ON THE LAST COLUMN
    row_group_order(
      groups = c("Unconditional response patterns",
                 md("*k* = 1 Conditional response patterns"),
                 md("*k* = 2 Conditional response patterns"),
                 md("*k* = 3 Conditional response patterns"))) %>%
    tab_footnote(
    footnote = html(
      "<i>Note.</i> <i>f</i>_r = response pattern frequency; P_{<i>k</i>} =
posterior class probabilities"
)
) %>%
  cols_align(align = "center") %>%
  opt_align_table_header(align = "left") %>%
  gt::tab_options(table.font.names = "Times New Roman")

resp_table
```
````

Save table

````
```{r, eval=FALSE}
gtsave(resp_table, here("figures","resp_table.png"))

```
````

````

Classification Diagnostics

Use Mplus to calculate k-class confidence intervals (Note: Change the synax to make your chosen *k*-class model)
```{r, eval=FALSE}
classification  <- mplusObject(

  TITLE = "C3 LCA - Calculated k-Class 95% CI",

  VARIABLE =
    "categorical = report_dis-law_fte;
   usevar =  report_dis-law_fte;
   classes = c(3);",

  ANALYSIS =
    "estimator = ml;
    type = mixture;
    starts = 0;
    processors = 10;
    optseed = 802779;
    bootstrap = 1000;",

  MODEL =
    "
  !CHANGE THIS SECTION TO YOUR CHOSEN k-CLASS MODEL

  %OVERALL%
  [C#1](c1);

  [C#2](C2);

  Model Constraint:
  New(p1 p2 p3);

  p1 = exp(c1)/(1+exp(c1)+exp(c2));
  p2 = exp(c2)/(1+exp(c1)+exp(c2));
  p3 = 1/(1+exp(c1)+exp(c2));",


  OUTPUT = "cinterval(bcbootstrap)",

  usevariables = colnames(df_bully),
  rdata = df_bully)

classification_fit <- mplusModeler(classification,
                dataout=here("mplus", "bully.dat"),
                modelout=here("mplus", "class.inp") ,
                check=TRUE, run = TRUE, hashfilename = FALSE)
```

Note: Ensure that the classes did not shift during this step (i.g., Class 1 in the enumeration run is now Class 4). Evaluate output and compare the class counts and proportions for the latent classes. Using the OPTSEED function ensures replication of the best loglikelihood value run.

Read in the 3-class model
```{r}
# Read in the 3-class model and extract information needed
output_bully <- readModels(here("mplus", "class.out"))

# Entropy
entropy <- c(output_bully$summaries$Entropy, rep(NA,
output_bully$summaries$NLatentClasses-1))

# 95% k-Class and k-class 95% Confidence Intervals
k_ci <- output_bully$parameters$ci.unstandardized %>%
  filter(paramHeader == "New.Additional.Parameters") %>%
  unite(CI, c(low2.5,up2.5), sep=", ", remove = TRUE) %>%
  mutate(CI = paste0("[", CI, "]")) %>%
  rename(kclass=est) %>%
  dplyr::select(kclass, CI)

# AvePPk = Average Latent Class Probabilities for Most Likely Latent Class Membership (Row)
by Latent Class (Column)
avePPk <- tibble(avePPk = diag(output_bully$class_counts$avgProbs.mostLikely))

# mcaPk = modal class assignment proportion
mcaPk <- round(output_bully$class_counts$mostLikely,3) %>%
  mutate(model = paste0("Class ", class)) %>%
  add_column(avePPk, k_ci) %>%
  rename(mcaPk = proportion) %>%
  dplyr::select(model, kclass, CI, mcaPk, avePPk)

# OCCk = odds of correct classification
OCCk <- mcaPk %>%
  mutate(OCCk = round((avePPk/(1-avePPk))/(kclass/(1-kclass)),3))

# Put everything together
class_table <- data.frame(OCCk, entropy)
```

Now, use `{gt}` to make a nicely formatted table
```{r}
class_table <- class_table %>%
  gt() %>%
    tab_header(
    title = "Model Classification Diagnostics for the 3-Class Solution") %>%
    cols_label(
      model = md("*k*-Class"),
      kclass = md("*k*-Class Proportions"),
```

~~~
      CI = "95% CI",
      mcaPk = html("McaP<sub>k</sub>"),
      avePPk = md("AvePP<sub>k</sub>"),
      OCCk = md("OCC<sub>k</sub>"),
      entropy = "Entropy") %>%
    sub_missing(7,
            missing_text = "") %>%
    tab_footnote(
    footnote = html(
      "<i>Note.</i> McaP<sub>k</sub> = Modal class assignment proportion;
AvePP<sub>k</sub> = Average posterior class probabilities; OCC<sub>k</sub> = Odds of
correct classification; "
    )
  ) %>%
  cols_align(align = "center") %>%
  opt_align_table_header(align = "left") %>%
  gt::tab_options(table.font.names = "Times New Roman")

class_table
```

Save table
```{r, eval=FALSE}
gtsave(class_table, here("figures","class_table.png"))

```
~~~

References

Hallquist, M. N., & Wiley, J. F.
(2018).
MplusAutomation: An R Package for Facilitating Large-Scale Latent Variable Analyses in Mplus.
Structural equation modeling: a multidisciplinary journal, 25(4), 621-638.

Muthén, B. O., Muthén, L. K., & Asparouhov, T.
(2017).
Regression and mediation analysis using Mplus.
Los Angeles, CA: Muthén & Muthén.

Muthén, L.K.
and Muthén, B.O.
(1998-2017).
Mplus User's Guide.
Eighth Edition.
Los Angeles, CA: Muthén & Muthén

R Core Team (2017).
R: A language and environment for statistical computing.
R Foundation for Statistical Computing, Vienna, Austria.

URL <http://www.R-project.org/>

Wickham et al., (2019).
Welcome to the tidyverse.
Journal of Open Source Software, 4(43), 1686, <https://doi.org/10.21105/joss.01686>

```
![](figures/UCSB_Navy_mark.png){width="75%"}
```
